# Disparities in delivery of ophthalmic care; An exploration of public Medicare data

**DOI:** 10.1371/journal.pone.0182598

**Published:** 2017-08-07

**Authors:** Cecilia S. Lee, Grace L. Su, Douglas M. Baughman, Yue Wu, Aaron Y. Lee

**Affiliations:** 1 Department of Ophthalmology, University of Washington, Seattle, Washington, United States of America; 2 Lewis Katz School of Medicine, Temple University, Philadelphia, Pennsylvania, United States of America; 3 Department of Ophthalmology, Puget Sound Veteran Affairs, Seattle, Washington, United States of America; Bascom Palmer Eye Institute, UNITED STATES

## Abstract

**Purpose:**

Cataract is a major cause of age-related eye diseases in the United States, and cataract extraction is the most commonly performed surgery on Medicare beneficiaries. Analyzing the pattern in delivery of cataract care at the national level can highlight areas of disparities. We evaluated geographic disparities seen in cataract surgery delivery to Medicare beneficiaries in the United States.

**Setting:**

Cataract extractions across the United States in 2012.

**Design:**

Cross-sectional study examining distance to provider and observed versus expected number of cataract extractions.

**Methods:**

Cataract extraction current procedural terminology codes were used to sum the total observed number of cataract extractions per cataract surgeon. Epidemiology data on expected number of cataract surgeries in one year by decade of life were extrapolated via a Gaussian Process model. A linear regression model was used to compare differences in delivery of care between US economic regions.

**Results:**

2.2 million patients underwent cataract surgery in the Medicare dataset in 2012. The average distance to the nearest provider was 9.846 miles (standard deviation: 11.410 miles). This distance was statistically significant (p < 2.0 x 10^−22^) in the New England (5.935 mi), Mideast (6.356 mi), Great Lakes (8.733 mi), Far West (9.038 mi), Southeast (9.793 mi), Southwest (12.711 mi), Plains (16.047 mi), and Rocky Mountain (17.934 mi) regions. The total number of expected cataract surgeries greater than 100 miles to the nearest cataract surgeon was 1,901, where Montana, South Dakota, and Texas each had over 200 of these expected distances.

**Conclusions:**

A large discrepancy exists in cataract delivery to the Medicare population based on geographic factors. Patients who live in rural areas travel farther on average to see ophthalmologists, resulting in a lower observed than expected rate of cataract surgery. Our results have implications in future allocation of resources and ophthalmologists.

## Introduction

Cataract is the most common cause of blindness in the world [1], and a major cause of age-related eye diseases in the United States (US). Cataract extraction is the most commonly performed intraocular surgery in the United States, and Medicare beneficiaries account for approximately 80% of all cataract surgeries performed in the US [2]. In the past 20 years, there has been a 2.5 fold increase in the number of cataract surgeries observed [2,3]. This number is expected to rise with advances in surgical technique, increasing demands in bilateral surgery, and reductions in the visual impairment threshold for cataract extraction [2,3].

Corrected vision from cataract surgery can lead to significant improvements in one's cognitive ability, capability to perform daily activities of living, burden to the family, and overall quality of life [4]. A routine ophthalmologic examination is essential for timely diagnosis and optimal treatment of ocular diseases [5]. However, access to ophthalmic care may be limited in different parts of the country. Patients with a lower socioeconomic status who report an age-related eye disease are significantly less likely to visit an eye-care provider, suggesting that there is a strong influence of socioeconomic circumstance on one's ocular health [5]. Varying ophthalmologist availability across the United States also contributes to the disparity seen as some patients are less likely to seek eye-care if proximate access is a barrier [6].

Demographic factors have been shown to influence the incidence of cataract and delivery of ocular care. Cataract progression is an age-related disease, and as expected, an increased number of observed cataract surgeries is seen in elderly patients [7]. In addition, a higher incidence of cataract has been shown in women than in men, after accounting for age [8]. Furthermore, the Baltimore Eye Survey found that untreated cataracts accounted for 27% of all blindness among black Baltimore residents, suggesting a disparity in cataract surgery utilization and under-treatment in black Medicare beneficiaries [9]. Therefore, many factors must be taken into account when assessing cataract incidence and subsequent treatment.

On April 9, 2014, the Centers for Medicare & Medicaid Services (CMS) released the Medicare Provider Utilization and Payment Data with information on services and procedures provided to all Medicare beneficiaries. This public data covers the 2012 health expenditures on the Medicare population for 100% of fee-for-services, which includes nearly 10 million records related to a total of $77 billion in Medicare payments [10,11]. Combining Medicare claims data with the US Census information can illustrate the nationwide demand and availability of cataract care delivery. In this study, we analyzed the Medicare administrative data with the US Census results to identify the number and geographic distribution of ophthalmologists delivering cataract extraction in the United States and evaluated the distance to providers for each region as well as utilization disparities that may exist in various regions.

## Methods

This was a cross-sectional study based on two publically available sources: 2012 Medicare Provider Utilization and Payment Data released by the Center for Medicare and Medicaid Services (CMS) (https://data.cms.gov) (https://www.cms.gov/Research-Statistics-Data-and-Systems/Statistics-Trends-and-Reports/Medicare-Provider-Charge-Data/Physician-and-Other-Supplier2012.html) and the 2010 United States Census Bureau summary file (https://www.census.gov/prod/cen2010/doc/sf1.pdf). The summed total of observed number of cataract extractions performed on Medicare beneficiaries for each cataract surgeon was determined using CPT codes (66984,66983, 66982) linked to national provider identifiers (NPI). The provider location was determined from the practice address listed for an NPI in the CMS data. The address was geocoded to translate an address into a GPS coordinate using Google Maps API.

**Fig 1:**
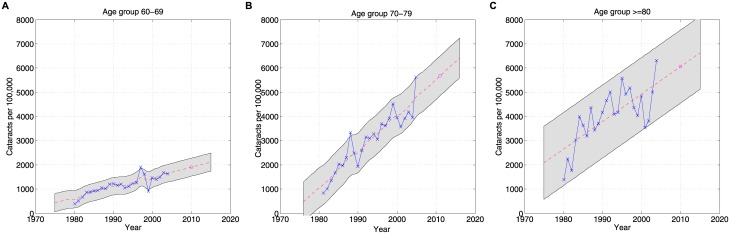
Predicted cataract rates using observed epidemiological data by decade of life using Gaussian Process regression. The dashed red lines represent the mean predictions. The shaded area represents the 95% confidence intervals, and the red circle represents the predicted cataract rates for 2010.

Epidemiologic data on expected number of cataract surgeries in one year by decade of life was extrapolated using a Gaussian Process model with data from previous literature [12]. Our study calculated the expected incidence of cataract surgeries in the US based on the age-specific cataract incidence found in Olmsted county, MN from 1980 to 2004 [12] (Fig 1).

In brief, Gaussian Process regression generalizes linear regression in two ways. First, it extends linear regression, which determines a linear functional relationship between the dependent variable, y, and the feature or covariate matrix, X. The feature matrix, X, is a transformation of the raw input data, Z. The main advantage of Gaussian Process regression over linear regression is that no pre-determined and handcrafted feature matrix needs to be constructed from the raw data. Handcrafting feature matrix is laborious, and one can never be sure that the ‘right’ features are included. Instead, Gaussian Process regression determines the functional relationship between y and Z using kernels or similarity functions. The intuition is that if two observations Z_1_ and Z_2_ are close, then their mappings into output space, y_1_ = f(Z_1_) and y_2_ = f(Z_2_) should be close as well. Second, the use of kernels in Gaussian Process regression yields more logical confidence intervals compared to linear regression. The confidence intervals in Gaussian Process regression are tight when there are many observations in a neighborhood and wider when there are fewer observations. For our analysis of cataract rates, we used Gaussian Process regression with squared exponential kernels. The kernel parameters of this Gaussian Process were learnt using gradient descent. More details on Gaussian Process regression are provided in the supplementary materials (S1 File).

Using the US census population estimates (total number and age) of each census block, we projected the expected number of cataract surgeries in each region. A hexagon layer was created to geographically normalize the US Census data. Each hexagon was sized with side length set to 0.005 GPS degrees, resulting in a unit area of 0.155 square miles (0.400 square kilometers). We hereafter refer to such unit areas as a normalized geographic unit (NGU). The Haversine distance function was used to calculate the distance from the center of each NGU to the nearest cataract provider. Provider location was mapped using red dots and the distance to the nearest provider for each NGU was represented with a line.

The ratio of expected vs. observed number of surgeries for each NGU within a provider’s 50-mile-radius was calculated as follows. The total number of cataract surgeries performed by that provider was divided by the number of NGUs within a 50 mile radius, giving an estimate for observed number of surgeries per NGU for that providers’ surrounding area. This number served as the denominator in our ratio. The expected number of surgeries per NGU was used as the numerator. For these areas, NGUs with ratios greater than one were shaded red (fewer surgeries than expected) and NGUs with ratios less than one were shaded blue (more surgeries than expected). NGUs with a positive number of expected cataracts that were located further than 50 miles to the nearest cataract surgeon were shaded deep red.

United States Bureau of Economic Analysis economic regions (bea.gov/regional/docs/regions.cfm) were used for regional analysis, with the exception that the Far West region excluded Alaska and Hawaii, as this was an analysis of the contiguous United States. Aunivariate linear regression model was used to compare distance to provider across 8 US regions (New England, Mideast, Southeast, Great Lakes, Far West, Southwest, Rocky Mountain, and Plains areas). State based totals for expected number of cataract surgery patients living >100 miles from the nearest provider were calculated. Multiple comparison adjusted p values were obtained using Tukey HSD methods for post-hoc pairwise comparisons. Finally, the observed and expected cataract surgery rates were summed for 50 square mile areas. All analyses were performed with Python, PostGIS, MySQL, and R (https://www.r-project.org/).

## Results

**Fig 2:**
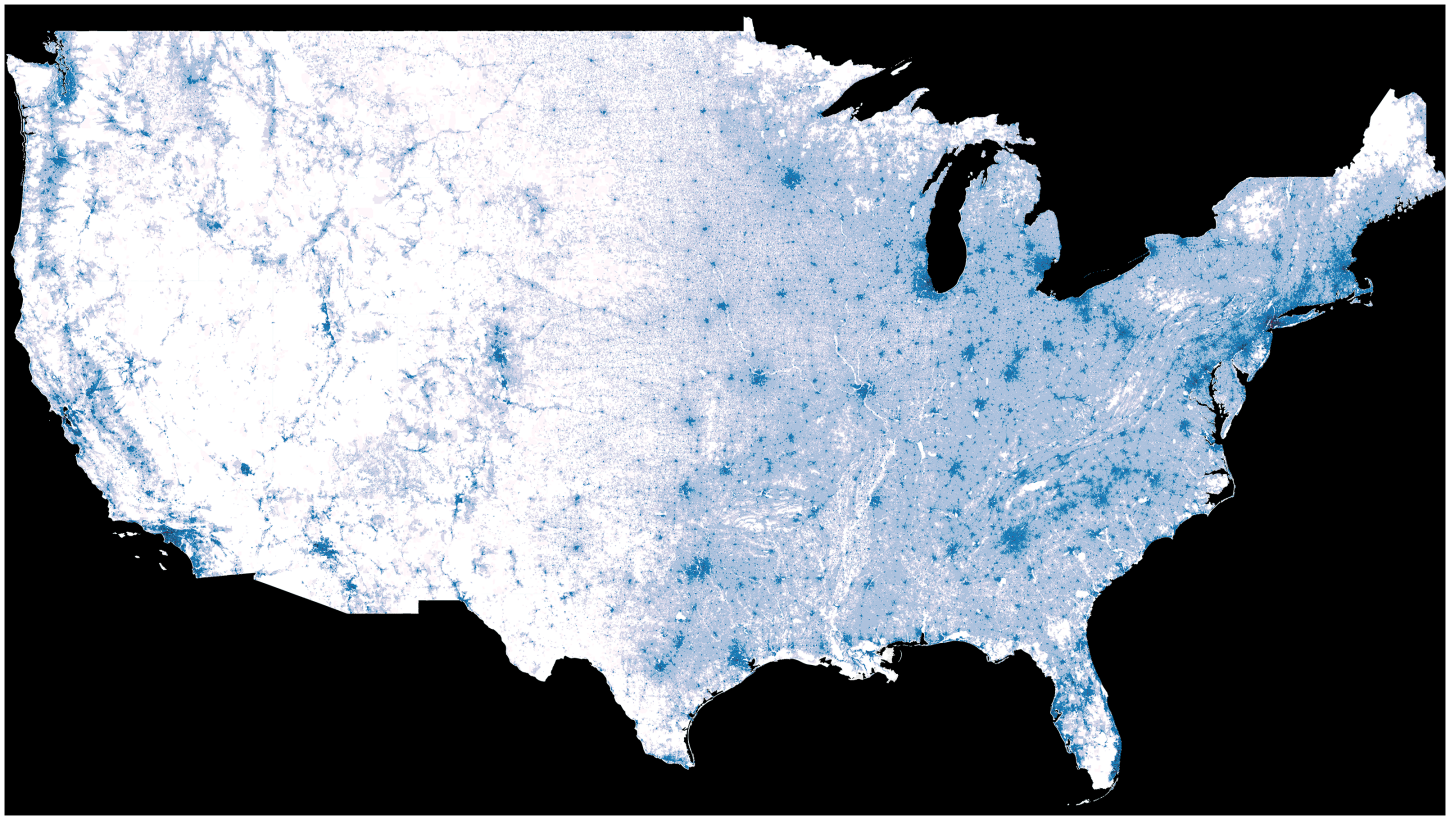
Choropleth of the continental US highlighting expected number of cataract surgeries. Darker blue and white areas represent a higher and an average density of expected cataract surgeries, respectively.

A total of 2.2 million Medicare patients underwent cataract surgery in 2012. Expected number of cataract surgeries per year was calculated based on the US census data for 2,214,034 distinct normalized geographical units (Fig 2).

**Fig 3:**
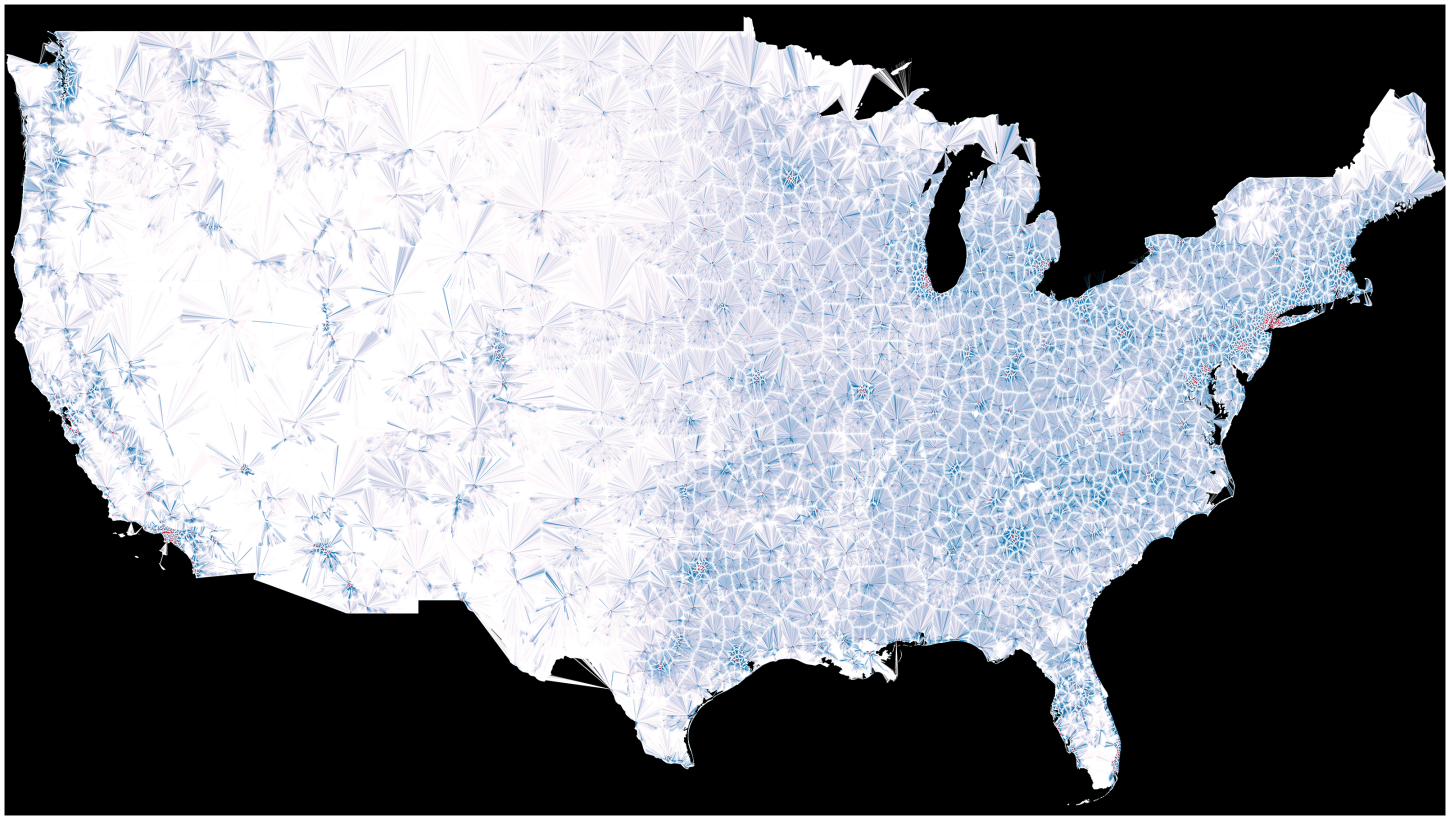
Choropeth of continental US highlighting distance to nearest surgeon. Each provider address is indicated by a red dot. A longer, darker line represents areas with higher than expected surgery rates living a greater distance from the nearest provider. White areas are comprised of these longer, darker blue lines.

**Fig 4:**
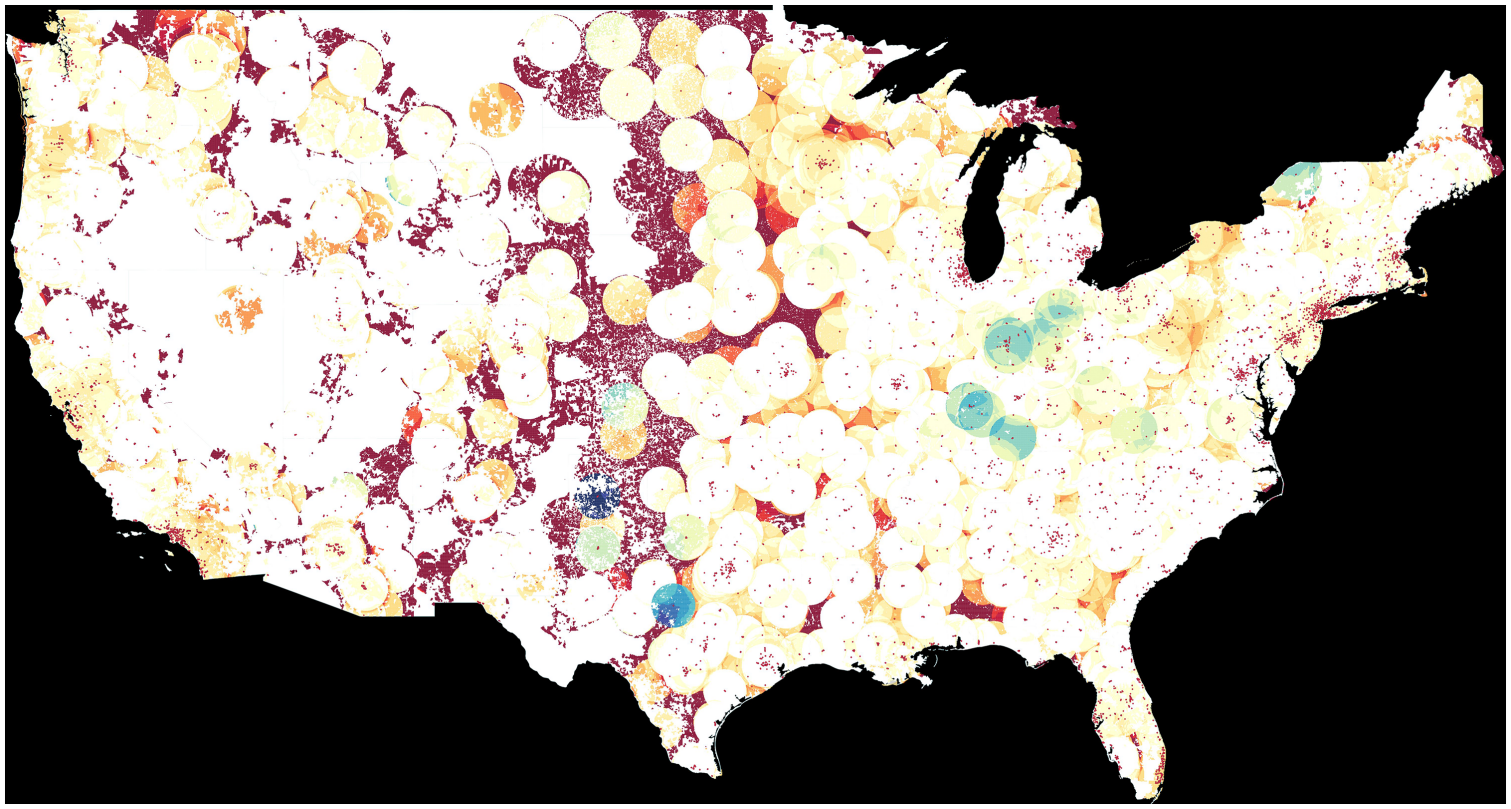
Choropleth of the ratio between observed vs. expected number of cataract surgeries. Provider addresses are indicated with a red dot. NGUs that fall within a 50 mile radius of a provider are shaded blue if their ratio shows more surgeries than expected and red if they show less surgeries than expected. White spots represent areas with an average number of expected vs. observed surgeries. NGUs falling outside a provider 50 mile radius are shaded deep red to indicate a very surgery-low area.

Distance to nearest cataract surgeon was calculated for each of the 2,214,034 locations and shown with overlaid line choropleth (Fig 3), and the expected vs. observed ratio choropleth is shown in Fig 4.

**Fig 5:**
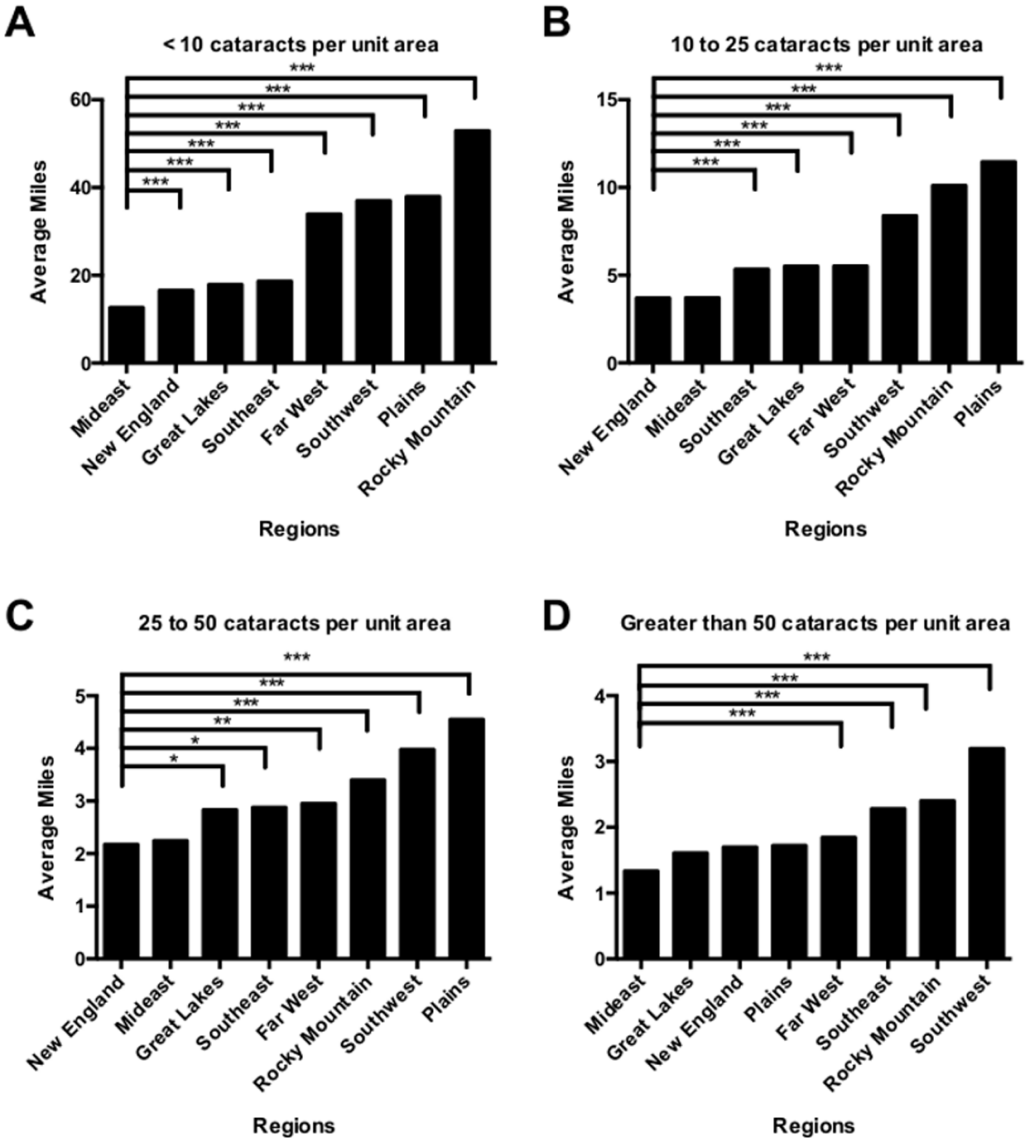
Average distance in miles to the nearest provider. Distance was stratified by expected number of cataracts per normalized geographic unit (NGU) with pairwise adjusted p values for multiple comparisons. Average distance for each region for <10 cataracts per NGU (A), 10 to 25 cataracts per unit area (B), 25 to 50 cataracts per unit area (C), and greater than 50 cataracts per unit area (D). * adjusted p value < 0.05, ** adjusted p value 0.01 to 0.05, adjusted p value < 0.001.

Quantitative analyses of choropleths in Figs 2–4 are summarized in Fig 5, stratified by the number of expected cataract surgeries per unit area. Southwest, Plains and Rocky Mountain regions had significantly higher average distance to the nearest cataract surgeon per NGU compared to other regions. The mean distance to the nearest cataract provider in the continental US was 9.846 miles (standard deviation: 11.410 miles). There was a statistically significant difference (p < 2.0 x 10^−22^) among the mean and standard deviations (SD) in the New England—6.154 miles (SD: 5.935 miles), Mideast—6.356 miles (SD: 5.284 miles), Great Lakes—8.733 miles (SD: 8.603 miles), Far West—9.038 miles (SD: 11.67 miles), Southeast—9.793 (SD: 8.654 miles), Southwest—12.711 miles (SD: 15.391 miles), Plains—16.047 miles), and Rocky Mountain—17.934 miles (SD: 24.375 miles) areas. Pairwise comparisons with adjusted p-values for multiple comparisons were stratified by differing ranges of expected cataracts per unit area and shown in Fig 5.

**Fig 6:**
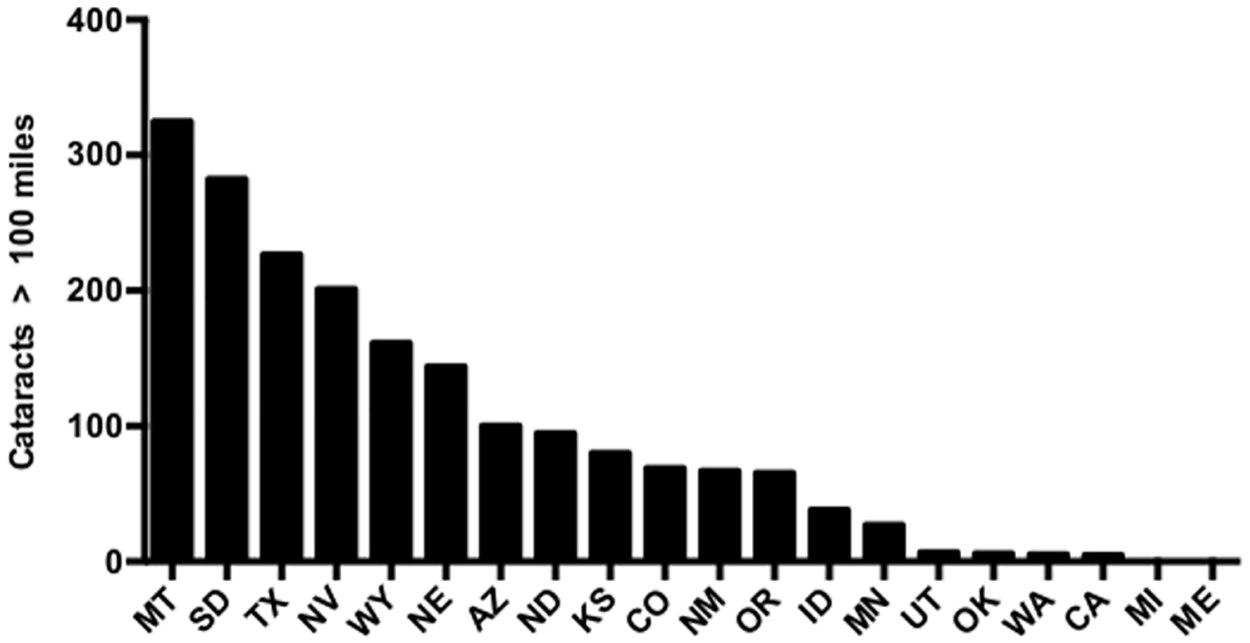
Annual expected number of cataract surgeries by state. Number of expected cataract surgeries greater than 100 miles from the nearest cataract surgeon's primary practice address.

The total number of expected cases of cataract surgeries greater than 100 miles to the nearest cataract surgeon was 1,901, and a rank order of these states were calculated (Fig 6). More than 100 expected surgeries at a greater than 100 miles to the surgeon were found in Montana (n = 324.82), South Dakota (282.27), Texas (226.60), Nevada (200.92), Wyoming (161.01), and Nebraska (142.91) (Fig 6).

**Fig 7:**
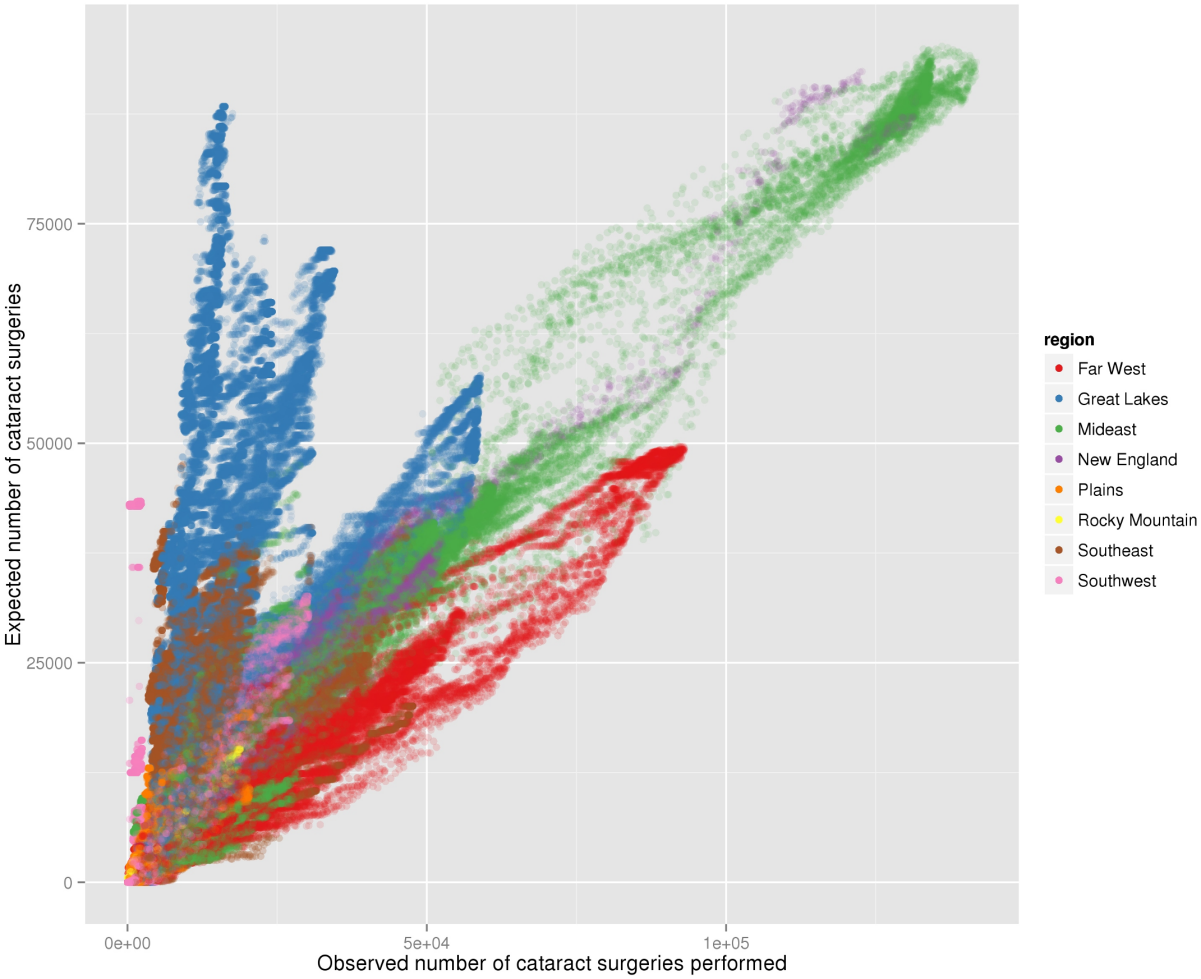
Observed vs. expected number of cataract surgeries. Scatterplot of 50 mile summed observed (y-axis) vs. expected (x-axis) number of cataract surgeries, color-coded by region.

A 50-mile average was calculated for expected number of cataract surgeries by population versus observed number of cataract surgeries, color-coded by US economic regions (Fig 7). The New England and Mideast areas had a roughly one-to-one ratio in expected versus observed cataract surgeries. The Great Lakes, Southeast, and Plains regions demonstrated more observed than expected surgeries, whereas the Far West area showed the opposite results (Fig 7).

## Discussion

In this study, we have identified areas of greater travel burden to cataract providers and areas or regions with differences in utilization of cataract surgery. The Plains and Rocky Mountains showed longer mean travel distances compared to other regions. A significant number of expected cataract patients resided at greater than 100 miles from the nearest provider in Montana, South Dakota, Texas, Nevada, and Wyoming. Disparities were found between expected versus observed cataract surgery delivery across the country, in particular the Plains and Rocky Mountains regions. Many of these utilization disparities were observed in regions with a lower density of ophthalmologists. On the other hand, New England and the Mideast had a higher density of ophthalmologists and a matched ratio in expected versus observed cataract surgeries. The Great Lakes had a high density of ophthalmologists and high utilization compared to the Mideast and Far West.

Previous studies have primarily focused on the demographic variations that areassociated with cataract care delivery disparities. Our data did not include demographic data; however, factors such as age [7], gender [8], and race [9] have been shown to influence the incidence of cataract and delivery of ocular care. In addition, a previous study examined the relationship between the latitude of residence and the likelihood of cataract extraction, hypothesizing that there would be an increased prevalence of cataract in warmer climate regions due to more exposure to ultraviolet-B from sunlight [7]. Furthermore, warmer climate might increase one's perceived visual needs due to more time spent engaged in outdoor activities. Latitude was found to have a 50% increase in cataract surgery in the southernmost regions of the United States [7]. However, our study detected similar discrepancies throughout the north and south and did not find latitude as a significant factor.

From previous research, travel burden is known to affect utilization in other areas of healthcare [13]. A study comparing the travel times, distances, and physician specialty mix of Medicare patients living in Alaska, Idaho, North and South Carolina, and Washington confirmed prior findings that rural residents have fewer overall clinic visits and see fewer medical specialists than their urban counterparts [6]. While patients may travel farther for life-threatening diseases such as cancer, they may not be as willing to travel long distances for elective surgical care such as cataract. In fact, another study examining the effect of travel distance on mammography facility choice found that women are less likely to seek mammograms, even if free of charge, if they must travel more than 20 miles [14]. Furthermore, the physician visit and compliance rate of elderly patients are much higher when practices are in close proximity to their other activities of daily living [15]. As a result, several state health departments have proposed a standard that residents should not have to travel more than 30 minutes to see a physician [16].

The current literature has not shown whether travel burden decreases utilization of cataract care [17]. In a study addressing travel burden and cataract care utilization, Kauh et al found that rural residence actually increased the chance of cataract surgery in diagnosed cataract patients within a nationwide managed care network between 2001 and 2011 [18]. Those living in rural communities had a 5% to 7% increased hazard of cataract surgery during that 10-year period, compared to those in urban communities. The authors attributed the increased likelihood to factors such as relying more heavily on better vision for driving the long distances to work and leisure activities, compared to urban areas with public transportation. However, this analysis did not account for patients with undiagnosed cataract. Thus, while rural patients with a diagnosed cataract may be more likely to undergo surgery, the burden of undiagnosed cataract left untreated in these areas may be significant, which was analyzed in our study.

Our results suggest that distance is an important marker for access to cataract care. The regions with the longest distance to provider also had the greatest utilization disparities, implying a negative association between travel burden and cataract care. While this finding falls in line with the widely accepted notion that distance decreases utilization [6,14], our analysis provides this finding specifically for cataract care with extensive geographic detail on distance to provider and utilization disparity across the United States.

Furthermore, our study reveals significant differences in utilization between regions. While utilization differences have been shown for other healthcare services [19], our analysis is unique in the combining of Medicare claims data and US census data. This allows for a thorough nationwide survey of cataract care delivery and access to care [20]. Interestingly, we identified the Great Lakes as a region of higher surgery rates compared to the Mideast and Far West (Fig 7), even though these three regions had similar average distances to provider. A possible explanation includes current fee-for-service Medicare reimbursement model used throughout the United States in which regional healthcare networks are incentivized to promote utilization of high-reimbursing procedures like cataract extractions leading to supply-driven demand [21]. With tools such as direct-to-patient advertising, healthcare corporations may produce regionally disparate patterns of patient care-seeking behavior [22]. In addition, regional provider attitudes may affect their recommendations to patients during the decision to undergo cataract surgery. Such differences in provider recommendation across regions have been seen for other surgical conditions [23].

Our study has several limitations. First, our analysis was cross-sectional and based solely on Medicare billing claims. Additional modifiers on claims or variability in billing practice due to Medicare coding regulations should be considered when evaluating the data. However, the Medicare part B database has been found to be 99% accurate in detecting cataract surgeries [24]. Second, our analysis did not include Alaska, a state with vast geographic territory and exceptionally long distances to providers in some areas. Inclusion of Alaska would have almost certainly changed the mean travel distance in the Far West region. Third, our choice of data source did not allow us to control for factors known to influence rates of cataract surgery, including race and sex. We were, however, able to account for age, an important factor affecting receipt of cataract care [12]. Fourth, the observed number of surgeries in each NGU was derived from data from Olmsted County, MN, where residents are less ethnically diverse and wealthier than the overall US population. As a result, the incidence may be different when generalizing to the rest of the US. However, the database includes subjects of all ages, and furthermore, a prior study [25] found that the overall annual incidence of hip fractures in Olmsted County (386/100,000) was similar to the 391/100,000 US rate, suggesting that results from this region have been generally consistent with national data. In addition, another study [26] that looked solely at Medicare beneficiaries from 2003 to 2004 reported a cataract incidence of 61.8/1000 person-years, with varying rates from 42/1000 in Wyoming to 86/1000 person-years in North Dakota. The authors also found lower rates among African Americans and higher rates in females, findings consistent with previous literature [8,9]. Thus, our estimates of 46.2/1000 person-years during the same time interval for people greater than 65 years of age are comparable but likely conservative. Lastly, we used 2010 census data that preceded CMS data by two years, and subtle population differences may be present.

Cataract extraction is a cost-effective surgery that has been shown to improve daily function, cognitive ability, and quality of life. Our results suggest that there is a large discrepancy in cataract care delivery based on geographic factors, which may lead to differing quality of care in areas with fewer ophthalmologists. This has significant implications on future allocations of resources including additional number of ophthalmologists. Further studies are warranted to generate ideas for more efficient, cost-effective healthcare delivery throughout the United States.

## Supporting information

S1 File Gaussian Process regression methodology. (PDF)
