# Supplementary material for: Disparities in delivery of ophthalmic care; An exploration of public Medicare data
Source: PLoS One. 2017 Aug 7;12(8):e0182598. doi: 10.1371/journal.pone.0182598 (PMC5546578; doi:10.1371/journal.pone.0182598)
Supplement: S1 File — (PDF) [file pone.0182598.s001.pdf]

1 Methodology

Gaussian Process Regression [1] is a generalization of linear regression. In standard linear regression, a linear relationship between a dependent variable y and a number of independent variables or covariates X is sought.

$$
\begin{aligned}
y &= X\beta + \epsilon \\
\epsilon &\sim \mathbb{N}(0, \sigma^2)
\end{aligned} \tag{1}
$$

The coefficients β are found by minimizing the squared loss:

$$
\hat{\beta} = \arg\min_{\beta} (X\beta - y)^2 \tag{2}
$$

$$
= (X^T X)^{-1} X^T y \tag{3}
$$

This is the Ordinary Least Squares (OLS) estimate. Moreover, theoretical confidence intervals and p-values can be found for both the coefficients β and predictions made using the coefficients. However, these theoretical values only hold if the conditions in equation (1) hold. Namely, y and X are Gaussian distributed and that their relationship is linear.

In most cases, the relationship between X and y will not be directly linear-Gaussian, and data transformations will be required. The transformations will try to translate the data so that:

1. The transformed dependent variable, y', is distributed according to a Gaussian distribution.

2. The transformed covariates, $\Phi(X)$, are distributed according to a multivariate Gaussian distribution.

3. The relationship between the transformed variables, y' and $\Phi(X)$, is close to linear.

Only when the above conditions are met, will the p-values and confidence intervals derived using OLS be meaningful. Otherwise, there is no guarantee that the p-values are correct.

Now let us consider regression in terms of these transformed variables. For the sake of simplicity of notation, we will assume in the rest of this document that the dependent variable y has already been transformed and is Gaussian distributed. Without loss of generality, when y is not Gaussian distributed, we can replace y in the following equations with a transformed y' that is Gaussian distributed. The equation for the regression on the transformed covariates is:

$$
\begin{aligned}
y &= \Phi(X)\beta + \epsilon \\
\epsilon &\sim \mathbb{N}(0, \sigma^2) \\
\hat{\beta} &= (\Phi(X)^T \Phi(X))^{-1} \Phi(X)^T y
\end{aligned} \tag{4}
$$

The equations (3) and (4) mirror each other with the variables X being replaced by their transformed counterparts $\Phi(X)$. Equation (4) is also called feature space regression [2], since regression was conducted on features were constructed from raw data. More importantly the feature matrix $\Phi(X)$ can contain any number of transformations of X. For example, it can include all powers of X if one is doing polynomial regression.

Since, the functional relationship between y and X is not known, it is desirable to make the feature matrix $\Phi(X)$ as rich as possible, so that the relationship can be found. However, this runs into two problems. First, if d, the number of dimensions of $\Phi(X)$ exceeds N, the number of data observations, i.e. $d > N$, $\Phi(X)^T\Phi(X)$ will be under-defined, as its rank is at most N. Then $(\Phi(X)^T\Phi(X))^{-1}$ will be singular as some eigenvalues will be zero. Second, even if $d \leq N$, one can easily overfit the data by including too many features in $\Phi(X)$. In this case, the weights $\hat{\beta}$ will minimize the equation (4) for the given data, $\{y_i, X_i\}_{i=1}^N$, but will make poor predictions for $y^\star$ when given a new covariate, $X^\star$.

A popular solution is to apply regularization. For example, with the common L2 regularization, the loss objective in equation (2) becomes:

$$\hat{\beta} = \arg\min_{\beta} \left[(X\beta - y)^2 + \lambda\beta^2\right] \tag{5}$$

This is also often called ridge regression. Then the maximum likelihood estimate for β is:

$$\hat{\beta} = (X^TX + \lambda I)^{-1}X^Ty \tag{6}$$

Here I is the identity matrix and λ is a parameter that controls the amount of regularization. Clearly in equation (6), $(X^TX + \lambda I)$ is invertible. Furthermore, the coefficients are squeezed towards 0. The result is that predictions using equation (6) have less error, as fewer spurious coefficients have any impact on predictions [3]. Here we applied regularization to the untransformed space. It is simple to apply regularization feature regression. As noted above, simply replace X by $\Phi(X)$ in equation (6).

The maximum likelihood solution of ridge regression in equation (6) is the same as the *maximum a posteriori* solution of Bayesian linear regression, though the motivation is different. A detailed discussion of Bayesian and classical philosophy is beyond the scope of this paper. However, we will note that the Bayesian framework is a full probabilistic model where everything is modeled as random variables. Specifically, the unknown coefficients β are considered random variables. On the other hand, the classical approach tries to learn the 'true' β from the data, and will converge to it given infinite data. Contrast the Bayesian setup below with the classical setup in equation (1):

$$\begin{aligned} y &= X\beta + \epsilon \\ \epsilon &\sim \mathbb{N}(0, \sigma^2) \\ \beta &\sim \mathbb{N}(0, \Sigma_0) \end{aligned} \tag{7}$$

We see the main difference is the introduction of a prior distribution for β with a mean of 0 and covariance of Σ_0. Applying Bayes' Rule, one can compute the posterior

distribution of the coefficients:

$$
\begin{aligned}
p(\beta|y, X) &= \frac{p(y|X, \beta)p(\beta)}{p(y|X)} \\
p(\beta|y, X) &\sim \mathbb{N}(\frac{1}{\sigma^2}A^{-1}Xy, A^{-1}) \\
A &= \frac{1}{\sigma^2}X^TX + \Sigma_0^{-1} \qquad (8)
\end{aligned}
$$

The main difference between the Bayesian and classical approaches is that the Bayesian posterior yields a distribution on the coefficients, whereas the classical method gives a point estimate.

Gaussian process regression is a generalization of Bayesian linear regression and feature regression. It extends feature regression by considering all mappings of $f(x)$, instead of just linear mappings on a pre-determined subset of feature transformations $\Phi(X)\beta$. We introduce the setup and some Gaussian Process notation below:

$$y = f(x) + \epsilon, \quad \epsilon \sim \mathbb{N}(0, \sigma^2) \qquad (9)$$

$$m(x) = E[f(x)] \qquad (10)$$

$$k(x, x') = E[f(x) - m(x)][f(x') - m(x')] \qquad (11)$$

$$f(x) \sim \mathbb{GP}(m(x), k(x, x')) \qquad (12)$$

Equation (12) simply expresses the mapping $f(x)$ as a distribution of the mean and covariance functions, equations (10) and (11) respectively. The covariance function computes how closely correlated variables x and x' are after being mapped. The intuition is that if variables x and x' are close to each other, then their respective mappings $f(x)$ and $f(x')$ should be close to each other.

As a practical example, we note that Gaussian Process Regression with a linear covariance function or kernel, equation (13), is mathematically equivalent to Bayesian regression described in equation (7):

$$k(x, x') = \sigma^2 + x \cdot x' \qquad (13)$$

The problem with the linear kernel is that it is rigid across all domains, even domains where there has been few data observations. This is inappropriate because for domains that have not been observed before, there is no guarantee that a mapping between y and X exists let alone be as tightly bound as suggested by a linear kernel.

To combat this we choose a more flexible kernel for our analysis. We used a squared exponential kernel:

$$k(x, x') = \exp\left(-\frac{(x - x')^2}{2l^2}\right) \qquad (14)$$

It is clear that for $(x - x') \rightarrow 0$, the covariance will be close to 1. On the other hand if $(x - x')^2$ is large, the covariance will approach 0 exponentially. The rate the covariance approaches 0 is controlled by the length scale parameter l. Large l mitigate large $(x - x')^2$, and make the rate of vanishing to zero lower. More importantly, large l will make covariances smoother locally.

For our study, the Gaussian Process mean function and covariance function, as well the length scale parameter l were learnt by performing gradient descent optimization. Then mean and 95% confidence interval predictions were made for a range of years for different age groups.

References

[1] C.E. Rasmussen and C.K.I. Williams. *Gaussian processes for machine learning*. Springer, 2006.

[2] C.M. Bishop et al. *Pattern recognition and machine learning*. Springer New York:, 2006.

[3] Trevor. Hastie, Robert. Tibshirani, and J Jerome H Friedman. *The elements of statistical learning*, volume 1. Springer New York, 2001.
